# Association of fruit and vegetable color with incident diabetes and cardiometabolic risk biomarkers in the United States Hispanic/Latino population

**DOI:** 10.1038/s41387-022-00197-0

**Published:** 2022-04-11

**Authors:** Zhiping Yu, Martha Tamez, Raymond Colon, Judith Rodriguez, Kristen K. Hicks-Roof, Nikki Ford, Josiemer Mattei, Daniela Sotres-Alvarez, Linda Van Horn, Matthew Allison, Gregory A. Talavera, Sheila F. Castañeda, Martha L. Daviglus

**Affiliations:** 1 Department of Nutrition and Dietetics, University of North Florida, Jacksonville, FL USA; 2 Department of Nutrition, Harvard T.H. Chan School of Public Health, Harvard University, Boston, MA USA; 3 Hass Avocado Board, Avocado Nutrition Center, Mission Viejo, CA USA; 4 Department of Biostatistics, Gillings School of Global Public Health, University of North Carolina at Chapel Hill, Chapel Hill, NC USA; 5 Department of Preventive Medicine, Feinberg School of Medicine, Northwestern University, Chicago, IL USA; 6 Department of Family Medicine, School of Medicine, University of California San Diego, San Diego, CA USA; 7 Department of Psychology, San Diego State University, San Diego, CA USA; 8 Institute for Minority Health Research, College of Medicine, University of Illinois at Chicago, Chicago, IL USA

**Keywords:** Nutrition, Epidemiology

## Abstract

**Background:**

Color groups of fruits and vegetables (FV) are part of a healthy diet, but evidence for an association with cardiometabolic outcomes is inconsistent.

**Objective:**

To examine the association between intake of FV of different colors with incident diabetes and cardiometabolic risk biomarkers among U.S. Hispanics/Latinos.

**Subjects/methods:**

We used data from 9206 adults ages 18–74 years who were free of diabetes at baseline (2008–2011) and had follow-up data at visit 2 (2014–2017) in the Hispanic Community Health Study/Study of Latinos (HCHS/SOL), a multicenter, prospective cohort study of self-identified Hispanics/Latinos. Dietary intake was assessed using two 24 h recalls at baseline. FV were categorized into five color groups: green, white, yellow/orange, red/purple, and uncategorized. Diabetes was defined based on laboratory measures and self-reported antihyperglycemic medication. We used survey logistic regression models to evaluate the association between FV color groups and incident diabetes and survey linear regression models to evaluate the association of FV color groups with cardiometabolic risk biomarkers at visit 2.

**Results:**

During ~6 years of follow-up, 970 incident cases of diabetes were documented. The red/purple FV color group was the least consumed (0.21 servings/day), whereas white FV were the most consumed (0.92 servings/day). For each serving of total FV intake, body mass index (BMI) was lower by 0.24% (*p* = 0.03) and insulin by 0.69% (*p* = 0.03). For each serving of red/purple FV intake, HDL was 1.59% higher (*p* = 0.04). For each serving of white FV intake (with potato), post-OGTT was 0.83% lower (*p* = 0.04) and triglycerides 1.43% lower (*p* = 0.04). There was no association between FV intake and incident diabetes.

**Conclusions:**

Specific FV colors were associated with cardiometabolic benefits though the associations were of relatively small magnitudes. Dietary recommendations could consider varying colors of FV intake, especially white and red/purple color groups, for a healthy diet.

## Introduction

According to American Diabetes Association (ADA), in 2019, 11.8% of Hispanics/Latios had diabetes with 8.3% among Central and South Americans, 6.5% among Cubans, 14.4% among Mexican Americans, and 12.4% among Puerto Ricans [1]. Diabetes is associated with an increased risk of many health problems such as heart disease, stroke, high blood pressure, and eye problems [2]. In addition, people suffering from diabetes bear a significant economic burden [3]. Compared to non-Hispanic Whites, Hispanics/Latinos are at a higher risk of developing diabetes [1, 4]. In 2017, diabetes was the 5th leading cause of death for Hispanic/Latino males and the 6th leading cause of death for Hispanic/Latino females [5]. According to the Centers for Disease Control and Prevention (CDC) this higher risk could be related to specific genes, higher obesity rates, lower levels of physical activity, cultural foods, and traditions [6].

Given the low fruit and vegetable (FV) consumption in the Hispanic/Latino population, the relationship between the consumption of different FV and diabetes may be relevant to diabetes risk [7, 8]. A meta-analysis of prospective cohort studies from 1966 to 2014 found that a higher intake of fruit (especially berries), green leafy vegetables, yellow vegetables, and cruciferous vegetables was associated with lower diabetes risk [9]. A review based on data from the Nurses’ Health Study and Health Professionals Follow-Up Study as well as a review of the biochemical properties of phenolic compounds helping hyperglycemia in diabetics have shown that diabetes risk differs by fruit varieties [10, 11].

Different arrays of nutrient profiles and specific phytochemicals may contribute to the mechanisms of diabetes and can distinguish fruit and vegetable color groupings. For example, anthocyanins contribute red, purple, and blue coloring to berries, grapes, tomatoes, and other fruits and vegetables. They have been associated with several potential health benefits, including anti-cancer, anti-inflammatory, anti-obesity, anti-diabetic, and cardiovascular disease prevention [12]. The carotenoids, highly present in yellow and orange or green FVs, are antioxidant and anti-inflammatory compounds associated with reduced risk of asthma, some cancers, and cardiovascular diseases [13, 14]. Although not studied extensively and more research is needed, data indicate that allium flavanols, common to white vegetables such as onions, have bioactive compounds with potential disease prevention through antioxidant and anti-inflammatory activity [15]. While some colorful FV compounds may appear to be more beneficial than others, the specific benefit may result from the amount of research and data rather than the biological effect of the colorful bioactive classification.

Existing research on FV color groups is limited, and results are mixed on the associations between FV color categories with metabolic and disease-related outcomes. For example, white FV consumption was inversely associated with 10-year stroke incidence in a Dutch prospective cohort study [16]. Similarly, a greater intake of orange/yellow, red/purple, and white vegetables was inversely associated with colorectal cancer risk in a Chinese population case-control study [17]. Other observational studies have found that a higher intake of red/blueberries, green leafy vegetables, and yellow vegetables is associated with lower diabetes risk [9, 18–21], but also a null association with green leafy vegetables and cruciferous vegetables in various ethnic groups [19, 22].

To date, most FV research was focused on specific FVs or FVs as a whole, rather than investigating the effect of different color groups on diabetes and no research has investigated this relationship in the United States (U.S.) Hispanic/Latino population. Thus, the present study aimed to determine if specific colors of FV are associated with incident diabetes and cardiometabolic risk biomarkers among U.S. Hispanic/Latino adults.

## Participants and methods

### Study design and population

The Hispanic Community Health Study/Study of Latinos (HCHS/SOL) is a multicenter, population-based cohort study of 16 415 men and women who self-identified as Hispanic or Latino (Mexican, Cuban, Puerto Rican, Dominican, Central, and South American), were age 18–74 years at recruitment, and lived in households selected at random in four U.S. field centers (San Diego, CA; Chicago, IL; Miami, FL; and Bronx, NY). Recruitment involved a stratified 2-stage area probability sample of addresses in each field center from 2008–2011 [23]. The sample design and cohort selection have been previously described [24]. Briefly, a stratified two-stage area probability sample of household addresses was selected in each of the four field centers. The first sampling stage randomly selected census block groups with stratification based on Hispanic/Latino concentration and proportion of high/low socio-economic status. The second sampling stage randomly selected households, with stratification, from US Postal Service registries that covered the randomly selected census block groups. Both stages oversampled certain strata to increase the likelihood that a selected address yielded a Hispanic/Latino household.

After households were sampled, in-person or telephone contacts were made to screen eligible households and to roster their members. Lastly, the study oversampled the 45–74 age group (*n* = 9714, 59.2%) to facilitate the examination of target outcomes. As a result, participants included in HCHS/SOL were selected with unequal probabilities of selection, and these probabilities need to be taken into account during data analysis to appropriately represent the target population. HCHS/SOL sampling weights are the product of a “base weight” (reciprocal of the probability of selection) and three adjustments: (1) non-response adjustments made relative to the sampling frame, (2) trimming to handle extreme values (to avoid a few weights with extreme values being overly influential in the analyses), and (3) calibration of weights to the 2010 U.S. Census according to age, sex, and Hispanic background.

All participants provided written informed consent. There were 11,623 participants who completed the follow-up visit from 2014–2017.

The institutional review boards of each field center, coordinating center, central lab, reading centers, and the National Heart, Lung, and Blood Institute approved this study. The study was registered at clinicaltrials.gov as NCT02060344. This analysis was reviewed and approved by the University of North Florida Institutional Review Board (IRB).

### Data collection

Both baseline (2008–2011) and follow-up (2014–2017) in-person examinations included anthropometric measurements, urine and fasting blood sample collection, and interviewer-administered questionnaires on the participants’ language preferences. The questionnaire collected demographic and socio-economic information, health and medical history, access and use of health care, smoking status history, physical activity, and medications/supplement use. Details of study design and study procedures can be found elsewhere [23, 24].

### Dietary assessment

Dietary assessment was completed at baseline (2008–2011). Detailed methods for dietary data collection have been previously published [25]. Briefly, dietary intake was assessed using data from two interviewer-administered 24 h dietary recalls. The first 24 h recall was administered in-person at the baseline examination, and the second recall via telephone ~30 days after baseline. Data were collected using the multiple-pass method of the Nutrition Data System for Research software, which contains over 18,000 foods, 8000 brand-name products, and many Hispanic and Latino foods. The software provides values for 139 nutrients, nutrient ratios, food-group serving counts, and other food components.

In the 24 h recall data, FV intakes were recorded for each participant by day. Using the U.S. Department of Agriculture’s Food Patterns Equivalents Database: 2009–2010: Methodology and User Guide (FPED), the FV weights were converted to cups by one-cup serving equivalents in grams. For food items lacking an FPED serving size, the U.S. Food and Drug Administration (FDA) serving size was used to count as one serving (e.g., French fries). Each one-cup equivalent was converted to standard serving size. With two exceptions (raw, green leafy vegetables, and dried fruit) a standard serving of a fruit or vegetable was defined as being a ½ cup. One cup was considered a serving for raw, leafy green vegetables, and a ¼ cup was considered a serving for dried fruits. Criteria for excluded FV were as follows: serving size information from FPED or FDA data was not available, mixed dishes (e.g., chicken and vegetable soup), beans, sauces, condiments, seasonings, jelly/jam, chips, and no weighed amount for food item provided by the participant. Mixed fruits or vegetables, and derivatives of certain foods (e.g., French fries) were included in the uncategorized category. We categorized all FVs into one of five color groups and used the modified color classification system by Mirmiran et al. [26].

Table S1 shows the color grouping and FV included for each color group (e.g., green, yellow/orange, red/purple, white, and uncategorized). Fruits and vegetables were combined due to the similar nutrient profile and phytochemicals in the same color group of fruit and vegetables. There are some exceptions, e.g., potato and corn have more starch content, and avocado has more monounsaturated fatty acids (MUFA) than other food items. Thus, in sensitivity analysis, we excluded them from their respective FV color group. The daily average one-cup equivalent and daily average serving were calculated for each participant and categorized by color and total. In this analysis, we used the average of the two 24 h dietary recalls.

### Cardiometabolic risk biomarkers and type 2 diabetes definition

Previous studies have suggested a large variety of metabolic factors that are potentially involved in the pathophysiology of diabetes (e.g., body mass index (BMI), glycosylated hemoglobin (HbA1c), fasting glucose, 2 h oral glucose tolerance test (OGTT), insulin, high-density cholesterol (HDL-C), low-density cholesterol (LDL-C), total cholesterol, triglycerides (TG), systolic blood pressure (SBP), and diastolic blood pressure (DBP)) and, thus, were used for this analysis [27–29].

Participants were required to fast for at least 8 h before the visit. A Roche Modular P Chemistry Analyzer was used to analyze serum triglycerides, serum HDL-C, serum LDL-C, total cholesterol, and fasting plasma glucose using a hexokinase enzymatic method (Roche Diagnostics Corporation, Indianapolis, IN). A 2 h OGTT was performed during the in-person visit. HbA1c was measured in EDTA whole blood using a Tosoh G7 Automated HPLC Analyzer (Tosoh Bioscience Inc., San Francisco, CA). Blood pressure was measured in triplicate with an automatic sphygmomanometer after a quiet rest and was averaged. Height was measured to the nearest 1.0 cm and weight to the nearest 0.1 kg; BMI was calculated as weight in kilograms divided by height in meters squared. The following BMI categories were used based on the CDC cutoffs [30]: underweight <18.5 kg/m^2^, healthy weight 18.5–<25 kg/m^2^, overweight 25–<30 kg/m^2^, and obesity ≥30 kg/m^2^.

The American Diabetes Association criteria were used to define diabetes based on fasting plasma glucose (≥126 mg/dL), 2 h OGTT (≥200 mg/dL), HbA1c level (≥6.5%), or self-reported use of medications for diabetes in the last 4 weeks [31]. Incident diabetes was a new case identified at follow-up visit from participants who were free of diabetes at baseline.

### Covariates

All covariates were from baseline data and include participants’ self-reported information on age, sex, field center, Hispanic/Latino heritage, household income, education level, whether U.S. born, and years living in the U.S. The following baseline covariates were included in the models. Self-reported hours of physical activity using the Global Physical Activity Questionnaire [32] were converted into metabolic equivalents and categorized as low, moderate, or high levels as described previously [33]. Sedentary behavior was self-reported by sitting and reclining time on Global Physical Activity Questionnaire [32]. A comprehensive questionnaire gauged cigarette use history, smoking status categories include non-smokers (smoked < 100 cigarettes and no present use), former smokers (smoked > 100 cigarettes but no present use), and current smokers (smoked daily or on some days). Alcohol use level was classified as no current use, low-level use (<7 drinks /week for females; <14 drinks/week for males), and high-level use (7+ drinks/week for females; 14+ drinks/week for males).

### Statistical analysis

Among 11,623 participants who had follow-up data, we excluded those with a diagnosis of diabetes at baseline (*n* = 2 401) due to the possibility of FV intake being influenced by any medical intervention a patient may receive from their health care team [34–37]. We also excluded individuals without 24 h dietary recalls (*n* = 16) for this analysis. These exclusions resulted in a final analytical sample size of 9206 adults. Individuals missing ethnic backgrounds were combined with the “others/mixed” category.

Individuals were categorized into two levels according to the median intake for overall FV and for each FV color group. Differences in baseline sociodemographic characteristics, lifestyle, and dietary intake by the median of FV color groups were tested using linear survey regression. The intake of the different FV color groups by ethnic background was examined by one-way analysis of variance (ANOVA) followed by Tukey post-hoc test. Differences in energy and nutrient intake from different FV color groups were also tested using ANOVA with *p*-value adjusted by Bonferroni correction for multiple comparisons. We used linear survey regressions to evaluate the association of intake of FV color groups with nutrient intake from all foods and with cardiometabolic biomarkers at follow-up (BMI, HbA1c, fasting glucose, post-OGTT, insulin, HDL-C, LDL-C, total cholesterol, TG, DBP, and SBP). Cardiometabolic risk biomarkers were log-transformed due to skewed distributions. We also evaluated the association between intakes of FV color groups and incident diabetes at follow-up using logistic survey regression models. Sex-stratified analyses were performed in linear survey regression models and logistic survey regression models to test differences by sex.

Linear and logistic regression models were adjusted for total energy intake only (model 1) and adjusted for baseline age, sex, income, education level, whether U.S. born, years living in the US, medication use for hypertension and blood lipids, BMI (except when BMI was the outcome), field center, Hispanic/Latino heritage, smoking status, alcohol use level, physical activity level, sedentary behavior, the time between baseline and follow-up visit, total energy intake, polyunsaturated fatty acids (PUFA), *trans* fatty acids, whole grains, red and processed meats, and sugar-sweetened beverages (model 2). Furthermore, the final models were mutually adjusted for the other color groups for an individual color group.

In sensitivity analysis using model 2, we repeated the analysis for white, yellow/orange, and uncategorized FV, excluding potato from the white group, corn from the yellow/orange group, and potato salad from the uncategorized group, since they are rich in starch and have a high glycemic index. We also removed avocado from the yellow/orange group due to its higher MUFA content. Sensitivity analyses were also conducted to additionally adjust models for nutrient intake (e.g., fiber, vitamin C) to test their impacts on the reported association.

We used survey-specific procedures for all analyses to account for HCHS/SOL complex sampling design. SAS 9.4 (SAS Institute, Cary, NC) was used for all analyses, and a *p*-value of <0.05 was considered statistically significant.

## Results

### Baseline characteristics and incident diabetes

During ~6 years of follow-up, 970 incident cases of diabetes were identified. The sociodemographic characteristics, lifestyle, and dietary intake data of HCHS/SOL individuals by median intake of FV are presented in Table 1. Compared to individuals consuming below the median of FV, those consuming above the median of FV were older, less obese, shorter smoking length, less percentage of heavy alcohol users, not born in the U.S., and more likely to be from the San Diego field center. Conversely, among those above the median, there were more individuals with higher income and education levels, had been living in the U.S. less than 10 years, and had greater energy intake. A higher percentage of Mexicans had a FV intake above the median, while a lower percentage of Puerto Ricans had an intake above the median.

**Table 1:** Sociodemographic, health, and behavioral characteristics and dietary intake of Hispanic/Latino adults by median intake of total servings/day of fruit and vegetable: HCHS/SOL, 2008–2011.

		Overall	Below median	Above median	
			(<2.85 servings/day)	(≥2.85 servings/day)	*P*-value
	*n*	9206	4603	4603	
Age (y)		40.9 ± 0.3	39.3 ± 0.3	42.7 ± 0.4	<0.0001
BMI (kg/m^2^)		29.0 ± 0.1	29.2 ± 0.1	28.7 ± 0.1	0.002
Sex, %					
Female	5809	55.7	56.0	55.4	0.76
Male	3397	44.3	44.0	44.6	
Field center, %					
Bronx	2022	26.1	29.2	22.7	<0.0001
Chicago	2416	17.1	17.7	16.5	
Miami	2336	29.2	28.9	29.5	
San Diego	2432	27.6	24.3	31.3	
Yearly household income, %					
$10,000 or less	1218	12.6	14.9	10.0	<0.001
$10,001–$20,000	2650	28.3	29.0	27.6	
$20,001–$40,000	2987	30.9	29.6	32.4	
$40,001–$75,000	1222	14.0	12.3	15.9	
$75,001 or more	405	5.6	4.7	6.5	
Not reported	724	8.6	9.5	7.6	
Education level, %					
Less than high school	3197	29.7	32.7	26.4	<0.0001
High School or equivalent	2393	28.0	29.4	26.3	
>Higher school or equivalent	3599	42.3	37.8	47.2	
Not reported	17	0.1	0.1	0.1	
Hispanic/Latino heritage, %					
Central American	981	7.3	7.9	6.6	<0.0001
Cuban	1334	20.0	19.8	20.2	
Dominican	817	9.5	8.7	10.4	
Mexican	3803	39.7	36.8	42.9	
Puerto Rican	1307	14.2	18.1	10.1	
South American	682	5.5	4.6	6.5	
Others/Mixed	282	3.7	4.1	3.3	
U.S. born, %					
U.S. born	1563	21.6	26.9	15.9	<0.0001
Not U.S. born	7635	78.4	73.1	84.1	
Years living in the U.S., %					
≥10 years	6914	70.9	73.0	68.6	0.002
<10 years	2266	29.1	27.0	31.4	
Incident diabetes, %					
No incident diabetes	8236	91.3	92.0	90.6	0.09
Incident diabetes	970	8.7	8.0	9.4	
BMI, %					
<18.5 kg/m^2^ (underweight)	69	1.2	1.4	1.0	0.004
18.5–24.9 kg/m^2^ (healthy weight)	1899	22.8	21.5	24.1	
25–29.9 kg/m^2^ (overweight)	3648	38.8	37.3	40.4	
≥30 kg/m^2^ (obesity)	3574	37.2	39.8	34.5	
Physical activity level, %					
Inactive	2041	20.4	21.8	19.0	0.12
Low activity	1192	12.2	12.2	12.2	
Medium activity	995	10.8	10.3	11.3	
High activity	4943	56.6	55.7	57.6	
Alcohol use level, %					0.03
No current use	4680	47.8	47.8	47.7	
Low level	4064	46.6	45.7	47.7	
High level	449	5.6	6.5	4.6	
Cigarette pack years		4.6 ± 0.2	5.0 ± 0.3	4.2 ± 0.3	0.02
Energy intake (kcal/d)		1977 ± 9.8	1934 ± 13.1	2024 ± 13.0	<0.0001
PUFA (g/d)		15.4 ± 0.1	15.4 ± 0.1	15.5 ± 0.1	0.43
*trans* fatty acids (g/d)		2.7 ± 0.02	2.8 ± 0.03	2.7 ± 0.03	0.008
Whole grain (servings/d)		1.6 ± 0.04	1.5 ± 0.05	1.7 ± 0.05	<0.0001
Red/processed meat (servings/d)		1.0 ± 0.01	1.1 ± 0.01	1.0 ± 0.01	0.02
SSB’s (servings/d)		1.8 ± 0.02	1.9 ± 0.03	1.8 ± 0.03	0.20

Values are means ± SEs or percentage. All analyses were weighted to adjust for sampling probability of selection and non-response.

*HCHS/SOL* Hispanic Community Health Study/Study of Latinos, *BMI* body mass index, *PUFA* polyunsaturated fatty acids, *SSB* sugar-sweetened beverage.

### Intakes of different colors of fruit and vegetables

**Fig. 1:**
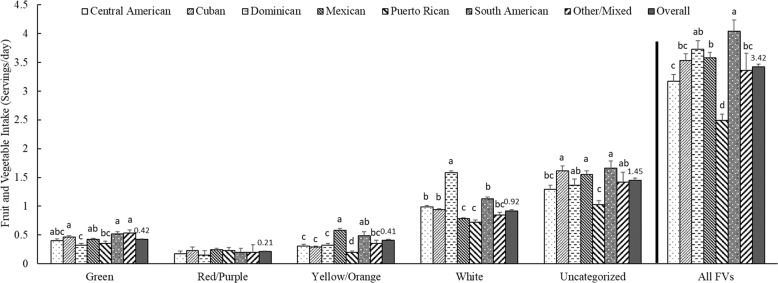
Mean fruit and vegetable intake (servings/day) by color group and Hispanic/Latino heritage in HCHS/SOL, 2008–2011. HCHS/SOL Hispanic Community Health Study/Study of Latinos. FV Fruit and vegetable. Values are weighted means ± SEs to adjust for sampling probability of selection and non-response. Different letters denote statistically significant differences among Hispanic/Latino heritage in each FV color group (green, red/purple, yellow/orange, white, uncategorized) and all FV color group.

We also analyzed the mean intake of FV color groups by Hispanic/Latino heritage (Fig. 1). Overall, the red/purple FV were the least consumed (0.21 servings/day), and uncategorized FV the most consumed (1.45 servings/day), followed by white FV (0.92 servings/day). Puerto Ricans had the lowest intake of FV, while South Americans consumed the highest FV (2.49 vs. 4.04 servings/day, respectively). The mean intake of green FV was highest among South Americans, Cuban, and other/mixed heritage, white FV among Dominicans, yellow/orange among Mexicans, red/purple was similar across all heritage groups, and uncategorized FV was highest among Cuban, Mexican, and South Americans.

### The association between fruit and vegetable intake and nutrients intake

The mean nutrient intakes from different FV color groups are shown in Table S2 and the associations between color FV intake and nutrient intake from all foods are presented in Table 2. Table S2 indicates that overall, each serving of white FVs contributed the highest energy and highest nutrients of total fat, saturated fatty acid, PUFA, carbohydrate, dietary fiber, vitamin B6, phosphorous, magnesium, and potassium. Each serving of green FVs contributed the highest MUFA, total protein, vitamin A, vitamin K, folate, calcium, iron, and zinc. Each serving of yellow and orange FVs contributed the highest MUFA, vitamin E, and vitamin C. Moreover (in Table 2); higher intakes of FV of all five color groups were associated with higher intake of dietary fiber, vitamin E, vitamin C, magnesium, and potassium and a lower intake of total saturated fat. Except for the red/purple group, higher intakes of all other four FV color groups were associated with higher intakes of vitamin A, and vitamin B6. In addition, higher consumption of specific color groups was associated with higher intakes of total energy (white, and uncategorized), carbohydrate (red/purple, yellow/orange, white, and uncategorized), protein (green, and uncategorized), PUFA (green), folate (green, yellow/orange, and white), and iron (green, and yellow/orange). Consumption of higher intakes of yellow/orange, white, and uncategorized color groups was associated with lower intakes of total fat, monounsaturated fat, and polyunsaturated fat. A higher intake of green FV was associated with lower intake of carbohydrates.

**Table 2:** Associations between intake of fruit and vegetable color groups and nutrient intake in HCHS/SOL, 2008–2011 (*n* = 9206)^a^.

ß coefficients	All colors^b^	Green^c^	Red/purple^c^	Yellow/orange^c^	White^c^	Uncategorized^c^
Energy (kcal)	20.9 ± 1.83***	−1.39 ± 6.49	7.52 ± 7.89	−0.52 ± 5.88	22.5 ± 3.77***	32.0 ± 3.36***
Total fat (g)	−0.57 ± 0.04***	0.19 ± 0.12	−0.44 ± 0.23	−0.62 ± 0.14***	−0.76 ± 0.08***	−0.64 ± 0.07***
Total SFA (g)	−0.25 ± 0.02***	−0.15 ± 0.07*	−0.29 ± 0.07***	−0.22 ± 0.07**	−0.31 ± 0.03***	−0.24 ± 0.03***
Total MUFA (g)	−0.20 ± 0.01***	0.15 ± 0.05**	−0.14 ± 0.08	−0.21 ± 0.06***	−0.26 ± 0.03***	−0.25 ± 0.03***
Total PUFA (g)	−0.09 ± 0.01***	0.17 ± 0.04***	0.04 ± 0.10	−0.19 ± 0.04***	−0.12 ± 0.03***	−0.12 ± 0.03***
Total carbohydrate (g)	1.86 ± 0.13***	−2.20 ± 0.42***	2.04 ± 0.49***	2.02 ± 0.44***	2.67 ± 0.24***	2.26 ± 0.21***
Total dietary fiber (g)	0.48 ± 0.03***	0.50 ± 0.06***	0.45 ± 0.11***	1.20 ± 0.07***	0.64 ± 0.04***	0.22 ± 0.04***
Total protein (g)	0.01 ± 0.05	1.47 ± 0.16***	−0.17 ± 0.35	−0.04 ± 0.12	−0.08 ± 0.09	−0.23 ± 0.07***
Vitamin A (mcg)	13.8 ± 0.98***	50.5 ± 2.85***	−3.30 ± 4.28	26.5 ± 3.37***	8.59 ± 1.79***	7.59 ± 1.68***
Vitamin E (IU)	0.18 ± 0.02***	0.45 ± 0.05***	0.23 ± 0.06***	0.34 ± 0.06***	0.06 ± 0.03*	0.15 ± 0.03***
Vitamin C (mg)	5.36 ± 0.24***	2.73 ± 0.45***	4.97 ± 1.06***	9.73 ± 0.62***	2.63 ± 0.28***	6.47 ± 0.53***
Vitamin B6 (mg)	0.03 ± 0.004***	0.04 ± 0.01***	0.01 ± 0.01	0.05 ± 0.01***	0.07 ± 0.01***	0.01 ± 0.00**
Folate (mcg)	4.14 ± 0.51***	13.38 ± 1.18***	2.49 ± 1.83	9.68 ± 1.40***	3.59 ± 1.00***	1.17 ± 0.80
Magnesium (mg)	5.27 ± 0.29***	7.61 ± 0.86***	3.99 ± 1.51**	9.18 ± 0.84***	5.84 ± 0.54***	3.61 ± 0.43***
Iron (mg)	0.05 ± 0.02**	0.18 ± 0.04***	0.11 ± 0.08	0.18 ± 0.07*	0.05 ± 0.03	−0.01 ± 0.02
Potassium (mg)	62.59 ± 1.88***	68.8 ± 5.09***	34.9 ± 13.9*	81.0 ± 5.95***	79.0 ± 3.25***	51.4 ± 3.3***

*HCHS/SOL* Hispanic Community Health Study/Study of Latinos, *SFA* saturated fatty acid, *MUFA* monounsaturated fatty acid, *PUFA* polyunsaturated fatty acid.

*p-value < 0.05; **p-value < 0.01; ***p-value < 0.001; *p*-values indicate the significance of association between each serving/day increase of F/V consumption and change of nutrients intake.

^a^Values are ß coefficients ± SEs for a one-serving/d increment.

^b^Models were adjusted for age, sex, Hispanic/Latino heritage, field center, physical activity, sedentary behavior, smoking, body mass index, income, education level, whether U.S. born, years living in the U.S., and total energy intake for nutrients.

^c^Models were adjusted for age, sex, Hispanic/Latino heritage, field center, physical activity, sedentary behavior, smoking, body mass index, income, education level, whether US born, years living in the U.S., and total energy intake for nutrients; furthermore, they were mutually adjusted by other color groups.

### The association between color fruit and vegetable intakes and cardiometabolic risk biomarkers at ~6 years follow-up

The association between baseline intake of FV color groups and cardiometabolic risk biomarkers~6 years later is shown in Table 3. After adjustment for confounders (model 2), for each serving of white FV intake (with potato), post-OGTT was on average lower by 0.83% and triglycerides by 1.43%. For each serving of red/purple FV intake, HDL was higher by 1.59%. For each serving of total FV intake, BMI was on average lower by 0.24% and insulin by 0.69%. None of the FV color groups were significantly associated with total cholesterol, LDL-C, SBP, DBP, HbA1c, or fasting glucose levels.

**Table 3:** Associations between intake of fruit and vegetable color groups at baseline and cardiometabolic risk biomarkers after ~6 years in HCHS/SOL, 2008–2011 to 2014–2017 (*n* = 9206)^a^.

(A) All analysis						
% change	All colors	Green	Red/purple	Yellow/orange	White	Uncategorized
Model 1—adjusted for total energy intake only^c^						
BMI^b^, kg/m^2^	−0.39 ± 0.10***	0.27 ± 0.36	−0.89 ± 0.40*	−0.31 ± 0.34	−0.47 ± 0.21*	−0.45 ± 0.18*
F-Glu^b^, mg/dL	0.10 ± 0.07	0.57 ± 0.26*	0.00 ± 0.30	0.10 ± 0.27	0.00 ± 0.18	0.06 ± 0.10
Post-OGTT^b^, mg/dL	0.45 ± 0.16**	0.54 ± 0.64	1.06 ± 0.82	1.01 ± 0.63	−0.15 ± 0.36	0.53 ± 0.26*
HbA1c^b^, %	0.10 ± 0.05*	0.26 ± 0.18	0.42 ± 0.19*	0.15 ± 0.16	0.13 ± 0.12	0.04 ± 0.07
Insulin^b^, pmol/L	−1.03 ± 0.32**	−0.61 ± 1.19	−3.15 ± 1.62	−0.35 ± 1.33	−1.70 ± 0.69*	−0.79 ± 0.50
LDL-C^b^, mg/dL	0.30 ± 0.13**	1.62 ± 0.60**	0.01 ± 0.81	−0.90 ± 3.10	−1.38 ± 1.36	−1.38 ± 3.13
HDL-C^b^, mg/dL	0.54 ± 0.16***	1.29 ± 0.51*	1.87 ± 0.82*	1.06 ± 0.72	0.85 ± 0.36*	−0.08 ± 0.25
TC^b^, mg/dL	0.31 ± 0.09***	1.20 ± 0.38**	0.71 ± 0.46	0.70 ± 0.33*	0.01 ± 0.23	0.18 ± 0.14
TG^b^, mg/dL	0.02 ± 0.31	−0.63 ± 1.04	0.03 ± 1.31	1.52 ± 1.27	−1.37 ± 0.70	0.56 ± 0.52
SBP^b^, mmHg	0.20 ± 0.07**	0.22 ± 0.24	0.20 ± 0.29	−0.12 ± 0.26	0.50 ± 0.14***	0.14 ± 0.13
DBP^b^, mmHg	−0.04 ± 0.09	−0.24 ± 0.29	0.06 ± 0.33	−0.76 ± 0.26**	0.26 ± 0.17	−0.01 ± 0.26

Model 2—adjusted for total energy intake and all other factors^d^						
BMI^b^, kg/m^2^	−0.24 ± 0.11*	0.42 ± 0.37	−0.72 ± 0.39	−0.02 ± 0.35	−0.22 ± 0.23	−0.39 ± 0.20
F-Glu^b^, mg/dL	0.04 ± 0.05	0.32 ± 0.25	−0.11 ± 0.27	−0.23 ± 0.27	0.15 ± 0.19	0.06 ± 0.11
Post-OGTT^b^, mg/dL	−0.24 ± 0.18	−0.18 ± 0.61	0.71 ± 0.81	−0.47 ± 0.65	−0.83 ± 0.40*	0.03 ± 0.28
HbA1c^b^, %	0.02 ± 0.05	0.19 ± 0.17	0.41 ± 0.22	0.01 ± 0.17	0.07 ± 0.12	−0.05 ± 0.07
Insulin^b^, pmol/L	−0.69 ± 0.32*	−0.78 ± 1.15	−2.27 ± 1.52	−0.66 ± 1.17	−0.37 ± 0.60	−0.63 ± 0.47
LDL-C^b^, mg/dL	−0.05 ± 0.15	0.85 ± 0.60	−0.52 ± 0.80	0.01 ± 0.50	−0.26 ± 0.32	−0.10 ± 0.23
HDL-C^b^, mg/dL	0.21 ± 0.16	0.73 ± 0.48	1.59 ± 0.80*	0.42 ± 0.75	0.37 ± 0.35	−0.21 ± 0.27
TC^b^, mg/dL	−0.03 ± 0.10	0.54 ± 0.38	0.28 ± 0.45	0.16 ± 0.35	−0.34 ± 0.23	−0.09 ± 0.15
TG^b^, mg/dL	−0.40 ± 0.33	−1.53 ± 1.05	−0.39 ± 1.30	0.59 ± 1.34	−1.43 ± 0.69*	0.14 ± 0.56
SBP^b^, mmHg	0.02 ± 0.08	−0.27 ± 0.21	−0.05 ± 0.25	−0.08 ± 0.22	0.16 ± 0.13	0.05 ± 0.13
DBP^b^, mmHg	0.02 ± 0.09	−0.44 ± 0.28	0.28 ± 0.33	−0.07 ± 0.28	0.07 ± 0.16	0.08 ± 0.15

(B) Sensitivity analysis using Model 2						
% change	Sensitivity analysis—no potato and corn^e^				Sensitivity ānalysis—no avocado^f^	
	All colors^c^	Yellow/ōrange^d^	White^d^	Uncategorized^d^	All colors^c^	Yellow/orange^d^
BMI^b^, kg/m^2^	−0.28 ± 0.11*	0.00 ± 0.35	−0.30 ± 0.23	−0.43 ± 0.20*	−0.23 ± 0.11*	0.11 ± 0.35
F-Glu^b^, mg/dL	0.06 ± 0.07	−0.19 ± 0.27	0.08 ± 0.20	0.07 ± 0.11	0.07 ± 0.07	−0.26 ± 0.28
Post-OGTT^b^, mg/dL	−0.15 ± 0.19	−0.51 ± 0.68	−0.68 ± 0.47	0.03 ± 0.29	−0.20 ± 0.18	−0.52 ± 0.69
HbA1c^b^, %	0.03 ± 0.05	0.06 ± 0.17	0.03 ± 0.12	−0.06 ± 0.08	0.05 ± 0.05	0.14 ± 0.18
Insulin^b^, pmol/L	−0.67 ± 0.33*	−0.44 ± 1.19	−0.33 ± 0.71	−0.63 ± 0.47	−0.72 ± 0.31*	−1.14 ± 1.17
LDL-C^b^, mg/dL	−0.03 ± 0.15	0.00 ± 0.52	−0.17 ± 0.34	−0.11 ± 0.24	−0.05 ± 0.15	0.09 ± 0.55
HDL-C^b^, mg/dL	0.19 ± 0.17	0.51 ± 0.78	0.29 ± 0.38	−0.23 ± 0.27	0.20 ± 0.16	0.25 ± 0.77
TC^b^, mg/dL	−0.00 ± 0.11	0.21 ± 0.37	−0.24 ± 0.25	−0.11 ± 0.15	−0.04 ± 0.10	0.17 ± 0.38
TG^b^, mg/dL	−0.29 ± 0.33	0.57 ± 1.38	−0.86 ± 0.78	0.05 ± 0.56	−0.41 ± 0.33	0.69 ± 1.38
SBP^b^, mmHg	0.02 ± 0.08	−0.10 ± 0.23	0.25 ± 0.14	0.03 ± 0.13	0.02 ± 0.08	−0.05 ± 0.25
DBP^b^, mmHg	0.01 ± 0.10	−0.12 ± 0.29	0.12 ± 0.18	0.06 ± 0.16	0.03 ± 0.10	0.02 ± 0.29

*HCHS/SOL* Hispanic Community Health Study/Study of Latinos, *BMI* body mass index, *F-Glu* fasting glucose, *OGTT* oral glucose tolerance test, *HbA1c* glycosylated hemoglobin, *LDL-C* low-density lipoprotein, *HDL-C* high-density lipoprotein, *TC* total cholesterol, *TG* triglycerides, *SBP* systolic blood pressure, *DBP* diastolic blood pressure.

*p-value < 0.05, **p-value < 0.01, ***p-value < 0.001.

^a^Values are percentage changes in cardometabolic risk biomarkers associated with one-serving increase in FV color groups.

^b^Cardiometabolic risk biomarkers were log-transformed due to skewed distributions and then back transformed to the estimates of coefficients on the original scale.

^c^Models were adjusted for total enery intake for all colors group. Furthermore, they were mutually adjusted by other color groups for individual color group.

^d^Models were adjusted for baseline data of age, sex, heritage, field center, income, education level, whether US born, years living in the U.S., medication use for hypertension and blood lipids, physical activity, sedentary behavior, smoking, alcohol use level, total energy intake, polyunsaturated fatty acids, *trans* fatty acids, whole grains, red and processed meat, sugar-sweetened beverage, time between baseline and follow-up visit, and BMI (except for BMI) for all colors group. Furthermore, they were mutually adjusted by other color groups for individual color group.

^e^Potato was excluded from the white group; corn was excluded from the yellow/orange group; potato salad was excluded from the uncategorized group; all three food items were excluded from the all colors group.

^f^Avocado was excluded from the yellow/orange group, and from the all colors group.

Results from sex-stratified analyses are presented in Table S3. In female participants only, for each serving of red/purple FV intake, BMI and TG were on average 2.04% and 3.68% lower, respectively, while HDL-C was on average higher by 2.90%. For each serving of total FV intake, insulin was on average lower by 1.10%, while HDL was higher by 0.42%. In male participants only, for each serving of white FV intake, TG level was lower by 2.21% and for each serving of uncategorized FV intake, BMI was lower by 0.49%.

In sensitivity analysis, when we excluded potatoes from the white group, corn from the yellow/orange group, and potato salad from the uncategorized group, the associations between white FV intake and OGTT and TG were no longer significant. However, we found a significant association between uncategorized FV intake and BMI: for each serving of uncategorized FV intake, BMI was on average lower by 0.43%. The associations between all FV intake and BMI and insulin remained significant (BMI was on average lower by 0.28% and insulin by 0.67%, respectively). In sensitivity analysis, when we excluded avocado from the yellow/orange group, the associations of all FV intake and yellow/orange FV intake with the cardiometabolic biomarkers remained the same.

### The association between color fruit and vegetable intakes and incident diabetes

The associations between each FV color group and incident diabetes are presented in Table 4 and Table S4. After adjusting for confounders (model 2) and in sensitivity analysis as well as sex-stratified analysis, we found no significant associations between intake of any FV color group and incident diabetes over ~6 years.

**Table 4:** Associations between intake of fruit and vegetable color groups and incident diabetes in HCHS/SOL, 2008–2011 to 2014–2017 (*n* = 9206).

Color groups	Incident diabetes^a^ OR (95% CI)	*P*-value
All analysis—Model 1^b^		
All colors	1.02 (0.99,1.06)	0.15
Green	1.06 (0.94,1.20)	0.36
Red/purple	1.00 (0.85,1.18)	0.97
Yellow/orange	0.99 (0.89,1.11)	0.89
White	1.04 (0.98,1.11)	0.18
Uncategorized	1.01 (0.96,1.07)	0.66
All analysis—Model 2^c^		
All colors	1.02 (0.98,1.06)	0.39
Green	1.07 (0.94,1.21)	0.31
Red/purple	1.01 (0.84,1.21)	0.90
Yellow/orange	0.96 (0.85,1.08)	0.49
White	1.03 (0.96,1.10)	0.45
Uncategorized	1.01 (0.95,1.08)	0.68
Sensitivity analysis: no potato and corn^d^		
All colors	1.02 (0.98,1.06)	0.36
Yellow/orange	0.96 (0.85,1.09)	0.55
White	1.03 (0.95,1.12)	0.46
Uncategorized	1.02 (0.96,1.08)	0.56
Sensitivity analysis: no avocado^e^		
All colors	1.02 (0.98, 1.06)	0.36
Yellow/orange	0.98 (0.87, 1.11)	0.77

*HCHS/SOL* Hispanic Community Health Study/Study of Latinos, *OR* odds ratio, *CI* confidence interval.

^a^The number of cases of incident diabetes was 970. Odds ratio and 95% confidence interval are for each serving of FV per day.

^b^Models were adjusted for total enery intake for all colors group. Furthermore, they were mutually adjusted by other color groups for individual color group.

^c^Models were adjusted for baseline data of age, sex, Hispanic/Latino heritage, field center, income, education level, whether U.S. born, years living in the U.S., medication use for hypertension and blood lipids, body mass index, physical activity, sedentary behavior, smoking, alcohol use level, total energy intake, polyunsaturated fatty acids, *trans* fatty acids, whole grains, red and processed meat, sugar-sweetened beverage, and time between baseline and follow-up visit for all colors group; furthermore, they were mutually adjusted by other color groups for individual color group.

^d^Potato was excluded from the white group; corn was excluded from the yellow/orange group; potato salad was excluded from the uncategorized group; all three food items were excluded from the all colors group.

^e^Avocado was excluded from the yellow/orange group, and from the all colors group.

## Discussion

This study assessed associations between intake of FV of different colors with incident diabetes and cardiometabolic risk biomarkers after ~6 years in a large U.S. cohort of Hispanics/Latinos. Consumption of total FV and specific colors of FV (particularly white color groups) was modestly associated with beneficial glycemic control and some cardiometabolic biomarkers ~6 years later.

Hispanics/Latinos consuming higher intakes of different color FV groups had a higher intake of fiber, vitamins, and minerals. Our findings with further adjustments (Table S5) suggested that these differences (especially fiber, magnesium, and potassium) may explain the observed associations with the cardiometabolic risk biomarkers. Dietary fiber is known to improve blood sugar levels and lower HbA1c and was associated with a lower risk of type 2 diabetes [9, 38, 39]. Magnesium has been shown to improve insulin resistance among individuals with diabetes [40]. A low level of potassium was associated with higher insulin levels and a higher risk of diabetes [41]. Other nutrients, bioactives, dietary components, or environmental variables that were not be able tested and considered cofounders in this analysis, may also have contributed to the observed results [42].

Although the magnitude was small, it is noteworthy that Hispanics/Latinos with higher white FV intake had lower levels of post-OGTT and triglycerides. This is consistent with the nutrient findings mentioned previously as the white FV group was associated with the highest intakes of fiber, magnesium, and potassium among all color groups. Overall, the white FV group was nutritionally diverse and included applesauce, apples, pears, bananas, plantains, and potatoes. Previous studies have shown that the consumption of apples and pears is associated with a lower risk of diabetes [43, 44]. Despite CDC recommendations to limit starchy vegetable intake [45], as well as previous research [46–49], researchers have called for the inclusion of white vegetables including potatoes to increase notably nutrients e.g., fiber, potassium, and magnesium, as well as increase overall vegetable consumption [50]. In those previous research, the positive associations between potato consumption and risk of diabetes were led by either high consumption of potatoes (e.g., one serving a day) [46–48] or the fried form (i.e., French fries) [48, 49]. It was also suggested that the glycemic index of white starchy vegetables using potato as an example, can be misleading if not interpreted in the context of the overall contribution that the white vegetable makes to the carbohydrate and nutrient composition of the diet [51]. As reported in a more recent study [49], our current study supports that potato and/or starchy vegetables in a non-fried form can also be part of a healthy diet that improves cardiometabolic risk biomarkers of diabetes in U.S. Hispanics/Latinos.

Red/purple FV intake was associated with a higher level of HDL-C in all participants, lower levels of BMI, TG, and higher HDL-C in female participants but not in males. Red/purple FVs are rich in polypheniols specifically anthocyanins. A prospective cohort reported that higher consumption of anthocyanin-rich foods was associated with a lower risk of type 2 diabetes [44]. In the same study, no significant associations were found for total flavonoid intake or other flavonoid subclasses [44]. In one clinical trial, berries—rich in anthocyanin—reduced postprandial insulin responses to bread in healthy women [52]. Our current findings in all participants and in female participants are consistent with those reports and many others [53, 54] suggesting that foods rich in anthocyanins may be one of the diet elements for the prevention and treatment of diabetes. No effects in males may suggest the sex difference as evidenced by males and females responding to diet and lifestyle modifications differently [55, 56]. More research may be needed to examine the effect of anthocyanins-rich foods on diabetes risk in males.

The green and the yellow/orange groups were not associated with any of the studied cardiometabolic risk biomarkers even though they were positively associated with the intake of many nutrients and contain high amounts of various nutrients themselves. In this study, the red/purple FV group includes fewer components and is consumed in less quantity, while the yellow/orange FV group has the greatest number of food components and is consumed in modest amounts. The diversity of the color group might contribute to the variance in response by groups if one food component has a more significant effect size than others.

Despite the possible advantages of consuming specific colors of FV on glycemic and other cardiometabolic markers, these benefits did not translate to lower odds of incident diabetes. This null association between FV intake and incident diabetes is consistent with the findings of some previous studies or systematic reviews [19, 22, 57]. However, in some other studies, the high consumption of FVs reduced incident diabetes in various populations [10, 58, 59]. In the current study, the average consumption of FVs was 3.42 servings/day, which is lower than the average FV consumption by the Americans (0.9 cups fruits/day and 1.4 cups vegetables/day, equals to 2.3 cups or roughly 4.6 servings FVs/day) [60], and far below the recommended 9 servings/day. The FV consumption in this population might be too low to show statistical effects on incident diabetes. Compared to those studies that reported a positive association, the relatively small sample size and shorter follow-up time (<10 years) of this study might also contribute to the null association. The null association is unlikely to be due to the sensitivity of diagnostic measures of diabetes since we used either fasting plasma glucose, post-OGTT, or HbA1c to minimize the misdiagnosis.

Although there is limited research on FV color groupings on health, dietary recommendations do have a partial basis in FV color. For example, the 2020–2025 Dietary Guidelines for Americans emphasize vegetables from specific color subgroups (e.g., dark-green and red/orange) to meet nutrient requirements [61]. One common approach to dietary advice is to have a “colorful plate”. The American Heart Association recommends “all the colors, all the time” to obtain all of the necessary vitamins, minerals, and nutrients [62]. The evidence seems to support this approach. A study of participants recording the color of the foods in their meals concluded that meal color variety was related to increased FV and a decreased intake of sugary foods [63]. In another experimental study, color variety modestly increased proximal intake, liking, and purchase intentions for fruits and vegetables in overweight or adults aged 36 years and older [64].

Our study has several strengths. The HCHS/SOL includes a large, diverse sample of Hispanics/Latinos recruited using probability sampling that provides an adequate representation of Hispanics/Latinos. We adjusted for several confounders that may influence the studied associations. The use of 24 h recalls for dietary assessment has been deemed useful in providing details about foods consumed, as well as culturally diverse foods [65]. A limitation of our study is that the study collected only two 24 h recalls at baseline only. The measurement error due to within-person variation may underestimate the magnitude of the observed associations. Although the extensive food preservation and transportation systems in the U.S. might have a relatively small contribution to variation in dietary intake, measurement error due to seasonal variations of available fruits and vegetables might be possible. This random measurement error could attenuate our results. It is also possible that participants’ dietary intake may have changed over time. Acquiring longitudinal dietary data in this population is essential to confirm the observed associations. Also, although categorizing FV by color is easily done for foods that are monochromatic and distinctive, classification is more complex and challenging to do when the definition is unclear of whether the color refers to the internal or external, or edible portion of the FV [26, 66, 67]. For example, spinach is easily discernible as green, but the color classification is not as straightforward for a red compared to a green apple or an avocado. Thus, the comparability of our results might be limited to studies that used the same FV classification. Although this simple color FV classification has not been validated yet, it has been used in multiple publications [26, 66, 67].

The results from our study support a growing body of literature to suggest that specific colors of FV differentially impact health. Regardless of the color, a high intake of FV contributes to meeting essential nutrient needs. Dietary recommendations for the Hispanic/Latino population could consider varying colors of FV, including the consumption of starchy vegetables in non-fried form, as part of a healthful diet to promote diabetes prevention.

## Supplementary information

Supplementary materials

## Data Availability

The datasets generated during and/or analyzed during the current study are available from the corresponding author on reasonable request.
