# Supplementary material for: Association of fruit and vegetable color with incident diabetes and cardiometabolic risk biomarkers in the United States Hispanic/Latino population
Source: Nutr Diabetes. 2022 Apr 11;12:18. doi: 10.1038/s41387-022-00197-0 (PMC9001729; doi:10.1038/s41387-022-00197-0)
Supplement: Supplementary file 1 — Supplementary materials [file 41387_2022_197_MOESM1_ESM.docx]

Table S1. Classification of fruit and vegetable by color group in HCHS/SOL, 2008-2011

| Green: 8 components   - Cooked greens (spinach, turnip, collard, mustard, chard, kale) - Raw greens (spinach, turnip, collard, mustard, chard, kale) - String beans/green beans - Peas - Broccoli - Lettuce salads (with or without other vegetables) - Dark green lettuce salads (spinach or romaine) - Nopal | White: 6 components^a^   - Applesauce - Apples - Pears - Bananas - Potatoes - Plantain |
| --- | --- |
| Yellow/Orange^a,b^: 11 components   - Pineapple - Peaches/nectarines/plums - Oranges/tangerines/clementines/tangelos - Mango - Papaya/lechosa/fruta bomba - Carrots - Corn - Summer squash (yellow and green squash) - Yams/sweet potatoes - Winter squash (pumpkin, acorn, butternut squash) - Avocado | Uncategorized: 7 components^a^   - Dried fruit (prunes, raisins) - Melons (cantaloupe, watermelon, honeydew) - Other fruit - Mix veggies - Potato salad - Salsa/pico de gallo - Other kind of vegetables |
| Red/Purple: 4 components   - Grapes - Strawberries - Red grapefruit - Tomato |  |

HCHS/SOL: Hispanic Community Health Study/Study of Latinos.

^a^An additional classification of white FV and yellow/orange FV were created excluding potato and corn due to high starch content. An additional classification of uncategorized FV was created excluding potato salad due to high starch content.

^b^An additional classification of yellow/orange FV was created excluding avocado due to high monounsaturated fatty acid content.

Table S2. Mean nutrient intake by fruit and vegetable color groups in HCHS/SOL, 2008-2011^a^

|  | Green  (n=11 523) | Red/Purple  (n=6 343) | Yellow/Orange  (n=10 488) | White  (n=14 871) | Uncategorized (n=33 770) | All  (n=76 995) |
| --- | --- | --- | --- | --- | --- | --- |
| Energy (kcal) | 39.86±0.66^d^ | 22.66±0.45^e^ | 67.04±0.66^c^ | 141.8±1.06^a^ | 74.28±0.54^b^ | 76.93±0.37 |
| Total fat (g) | 2.38±0.05^a^ | 0.16±0.003^d^ | 1.93±0.04^b^ | 2.46±0.05^a^ | 1.04±0.02^c^ | 1.56±0.02 |
| Total SFA (g) | 0.48±0.01^b^ | 0.03±0.0006^e^ | 0.32±0.008^c^ | 0.54±0.01^a^ | 0.19±0.005^d^ | 0.31±0.004 |
| Total MUFA (g) | 0.99±0.02^a^ | 0.03±0.002^d^ | 1.04±0.02^a^ | 0.73±0.02^b^ | 0.34±0.01^c^ | 0.58±0.007 |
| Total PUFA (g) | 0.74±0.02^b^ | 0.06±0.001^d^ | 0.33±0.01^c^ | 0.98±0.03^a^ | 0.39±0.01^c^ | 0.52±0.008 |
| Total carbohydrate (g) | 3.80±0.05^e^ | 5.46±0.12^d^ | 12.90±0.13^c^ | 31.63±0.20^a^ | 16.06±0.12^b^ | 15.93±0.07 |
| Total protein (g) | 1.57±0.03^a^ | 0.58±0.009^e^ | 1.23±0.01^c^ | 1.36±0.01^b^ | 1.05±0.008^d^ | 1.17±0.007 |
| Total dietary fiber (g) | 1.50±0.02^c^ | 0.89±0.012^e^ | 2.54±0.02^b^ | 3.50±0.02^a^ | 1.23±0.01^d^ | 1.86±0.008 |
| Vitamin A (IU) | 3130.3±43.6^a^ | 427.7±7.81^d^ | 2135.0±77.27^b^ | 383.7±6.60^d^ | 939.2±27.8^c^ | 1280.6±17.76 |
| Vitamin D(mcg) | 0.02±0.001^b^ | 0.0004±0.0003^c^ | 0.003±0.0005^c^ | 0.02±0.001^b^ | 0.04±0.002^a^ | 0.02±0.001 |
| Vitamin E (mg) | 0.61±0.009^b^ | 0.31±0.004^d^ | 0.69±0.009^a^ | 0.43±0.006^c^ | 0.45±0.006^c^ | 0.49±0.004 |
| Vitamin K(mcg) | 60.61±1.15^a^ | 5.26±0.09^c^ | 4.73±0.07^c^ | 3.91±0.007^c^ | 8.59±0.26^b^ | 14.67±0.22 |
| Vitamin C (mg) | 8.45±0.21^e^ | 14.38±0.26^c^ | 31.6±0.42^a^ | 11.7±0.08^d^ | 29.86±0.27^b^ | 22.11±0.14 |
| Vitamin B6 (mg) | 0.07±0.001^c^ | 0.05±0.001^d^ | 0.10±0.001^a^ | 0.32±0.002^b^ | 0.11±0.001^b^ | 0.14±0.001 |
| Folate (mcg) | 45.27±0.60^a^ | 9.33±0.13^e^ | 31.16±0.32^b^ | 21.57±0.18^c^ | 20.15±0.17^d^ | 24.80±0.13 |
| Calcium (mg) | 37.06±0.65^a^ | 8.51±0.14^d^ | 22.03±0.29^c^ | 10.57±0.14^d^ | 28.41±0.45^b^ | 23.75±0.23 |
| Phosphorous (mg) | 31.82±0.52^b^ | 15.95±0.20^d^ | 27.21±0.30^c^ | 37.20±0.33^a^ | 26.97±0.21^c^ | 28.80±0.14 |
| Magnesium (mg) | 14.22±0.21^c^ | 7.17±0.09^d^ | 15.62±0.14^b^ | 30.27±0.23^a^ | 14.81±0.10^c^ | 17.2±0.08 |
| Iron (mg) | 0.57±0.008^a^ | 0.21±0.003^e^ | 0.32±0.004^d^ | 0.51±0.006^b^ | 0.39±0.003^c^ | 0.41±0.002 |
| Zinc (mg) | 0.24±0.004^a^ | 0.10±0.001^d^ | 0.22±0.002^b^ | 0.23±0.003^b^ | 0.17±0.001^c^ | 0.19±0.001 |
| Potassium (mg) | 179.5±2.01^d^ | 145.3±1.76^e^ | 235.0±1.99^b^ | 453.4±3.37^a^ | 225.86±1.55^c^ | 257.4±1.09 |

HCHS/SOL, Hispanic Community Health Study/Study of Latinos; SFA, saturated fatty acids; MUFA, monounsaturated fatty acids; PUFA, polyunsaturated fatty acids.

^a^Values are weighted means/serving color FV ± SEs to adjust for sampling probability of selection and nonresponse.

^b^Different letters in each row denote the statistically significant differences among color groups for each nutrient. P-values were adjusted by Bonferroni corrections.

Table S3 Sex-stratified analysis for associations between intake of fruit and vegetable color groups at baseline and cardiometabolic risk biomarkers after ~6 years in HCHS/SOL, 2008-2011 to 2014-2017 ^a^

| % change | All colors^c^ | Green^d^ | Red/Purple^d^ | Yellow/Orange^d^ | White^d^ | Uncategorized^d^ |
| --- | --- | --- | --- | --- | --- | --- |
| Females (n=5 809) |  |  |  |  |  |  |
| BMI^b^, kg/m^2^ | -0.22±0.17 | -0.08±0.48 | -2.04±0.65** | 0.06±0.45 | -0.04±0.30 | -0.22±0.32 |
| F-Glu^b^, mg/dL | 0.11±0.10 | -0.02±0.29 | -0.41±0.42 | -0.03±0.28 | 0.33±0.26 | 0.13±0.14 |
| Post OGTT^b^, mg/dL | -0.28±0.19 | -0.57±0.71 | 0.25±1.14 | -0.39±0.70 | -0.57±0.44 | -0.12±0.34 |
| HbA1c^b^, % | 0.05±0.07 | -0.16±0.21 | 0.48±0.26 | -0.07±0.18 | 0.14±0.18 | 0.03±0.09 |
| Insulin^b^, pmol/L | -1.10±0.41** | -0.97±1.40 | -2.60±2.14 | -1.09±1.18 | -0.72±1.78 | -1.18±0.70 |
| LDL-C^b^, mg/dL | -0.31±0.20 | 0.77±0.76 | -0.65±0.91 | -0.39±0.65 | -0.21±0.45 | -0.60±0.37 |
| HDL-C^b^, mg/dL | 0.42±0.19* | 0.79±0.55 | 2.90±0.82*** | 0.75±0.66 | 0.20±0.41 | 0.07±0.32 |
| TC^b^ mg/dL | -0.18±0.13 | 0.48±0.50 | 0.09±0.60 | -0.16±0.43 | -0.16±0.43 | -0.42±0.22 |
| TG^b^, mg/dL | -0.76±0.36* | -1.77±1.36 | -3.68±1.70* | -0.46±1.33 | -0.40±0.82 | -0.47±0.60 |
| SBP^b^, mmHg | -0.05±0.08 | -0.29±0.30 | -0.34±0.38 | -0.07±0.25 | 0.10±0.17 | -0.03±0.14 |
| DBP^b^, mmHg | -0.11±0.09 | -0.64±0.40 | -0.03±0.51 | 0.08±0.33 | -0.06±0.21 | -0.06±0.15 |
| Males (n=3 397) |  |  |  |  |  |  |
| BMI^b^, kg/m^2^ | -0.25±0.14 | 0.92±0.53 | 0.11±0.51 | -0.14±0.51 | -0.39±0.32 | -0.49±0.24* |
| F-Glu^b^, mg/dL | 0.05±0.11 | 0.70±0.42 | 0.11±0.35 | -0.47±0.43 | 0.02±0.25 | 0.04±0.15 |
| Post OGTT^b^, mg/dL | -0.10±0.26 | 0.31±1.03 | 1.13±1.08 | -0.34±1.01 | -0.93±0.61 | 0.07±0.42 |
| HbA1c^b^, % | 0.03±0.07 | 0.55±0.28 | 0.33±0.31 | 0.12±0.30 | 0.00±0.15 | -0.11±0.10 |
| Insulin^b^, pmol/L | -0.34±0.46 | -0.79±1.72 | -2.68±2.37 | -0.28±2.07 | 0.12±0.93 | -0.15±0.60 |
| LDL-C^b^, mg/dL | 0.13±0.20 | 0.78±1.01 | -0.59±1.32 | 0.38±0.81 | -0.30±0.44 | 0.29±0.31 |
| HDL-C^b^, mg/dL | 0.05±0.25 | 0.77±0.81 | 0.80±1.20 | 0.16±1.19 | 0.40±0.54 | -0.40±0.41 |
| TC^b^, mg/dL | 0.07±0.14 | 0.56±0.65 | 0.28±0.73 | 0.49±0.56 | -0.52±0.31 | 0.18±0.21 |
| TG^b^, mg/dL | -0.08±0.53 | -1.36±1.66 | 1.50±1.93 | 1.67±2.11 | -2.20±1.11* | 0.69±0.84 |
| SBP^b^, mmHg | 0.09±0.12 | -0.25±0.27 | 0.02±0.31 | -0.14±0.37 | 0.17±0.19 | 0.16±0.20 |
| DBP^b^, mmHg | 0.14±0.15 | -0.17±0.41 | 0.42±0.43 | -0.09±0.45 | 0.18±0.23 | 0.19±0.24 |

HCHS/SOL, Hispanic Community Health Study/Study of Latinos; BMI, body mass index; F-Glu: fasting glucose; OGTT, oral glucose tolerance test; HbA1c, glycosylated hemoglobin; LDL-C, low-density lipoprotein; HDL-C, high-density lipoprotein; TC, total cholesterol; TG, triglycerides; SBP, systolic blood pressure; DBP, diastolic blood pressure.

^a^Values are percentage changes in cardometabolic risk biomarkers associated with one-serving increase in FV color groups.

^b^Cardiometabolic risk biomarkers were log-transformed due to skewed distributions and then back transformed to the estimates of coefficients on the original scale.

^c^Models were adjusted for baseline data of age, sex, Hispanic/Latino heritage, field center, income, education level, whether U.S. born, years living in the U.S., medication use for hypertension and blood lipids, physical activity, sedentary behavior, smoking, alcohol use level, total energy intake, polyunsaturated fatty acids, *trans* fatty acids, whole grains, red and processed meat, sugar-sweetened beverage, time between baseline and follow up visit, and BMI (except for BMI).

^d^Models were adjusted for baseline data of age, sex, heritage, field center, income, education level, whether US born, years living in the U.S., medication use for hypertension and blood lipids, physical activity, sedentary behavior, smoking, alcohol use level, total energy intake, polyunsaturated fatty acids, *trans* fatty acids, whole grains, red and processed meat, sugar-sweetened beverage, time between baseline and follow up visit, and BMI (except for BMI). Furthermore, they were mutually adjusted by other color groups.

Table S4. Sex-stratified analysis for associations between intake of fruit and vegetable color groups and incident diabetes in HCHS/SOL, 2008-2011 to 2014-2017

| Color groups | Incident diabetes^a^  OR (95% CI) | P-value |
| --- | --- | --- |
| Females (n=5 809) |  |  |
| All colors^b^ | 1.00 (0.96,1.05) | 0.94 |
| Green^c^ | 0.99 (0.85,1.15) | 0.90 |
| Red/Purple^c^ | 0.93 (0.68,1.27) | 0.65 |
| Yellow/Orange^c^ | 1.00 (0.87,1.16) | 0.95 |
| White^c^ | 1.05 (0.95,1.16) | 0.35 |
| Uncategorized^c^ | 0.98 (0.91,1.07) | 0.67 |
| Males (n=3 397) |  |  |
| All color^b^ | 1.03 (0.97,1.09) | 0.35 |
| Green^c^ | 1.15 (0.94,1.39) | 0.17 |
| Red/Purple^c^ | 1.08 (0.87,1.33) | 0.49 |
| Yellow/Orange^c^ | 0.90 (0.73,1.10) | 0.32 |
| White^c^ | 1.01 (0.91,1.12) | 0.90 |
| Uncategorized^c^ | 1.03 (0.95,1.12) | 0.43 |

HCHS/SOL, Hispanic Community Health Study/Study of Latinos; OR, odds ratio; CI, confidence interval

^a^The number of cases of incident diabetes was 970. Odds ratio and 95% confidence interval are for each serving of FV per day.

^b^Models were adjusted for baseline data of age, sex, Hispanic/Latino heritage, field center, income, education level, whether U.S. born, years living in the U.S., medication use for hypertension and blood lipids, body mass index, physical activity, sedentary behavior, smoking, alcohol use level, total energy intake, polyunsaturated fatty acids, *trans* fatty acids, whole grains, red and processed meat, sugar-sweetened beverage, and time between baseline and follow up visit.

^c^Models were adjusted for baseline data of age, sex, Hispanic/Latino heritage, field center, income, education level, whether U.S. born, years living in the U.S., medication use for hypertension and blood lipids, body mass index, physical activity, sedentary behavior, smoking, alcohol use level, total energy intake, polyunsaturated fatty acids, *trans* fatty acids, whole grains, red and processed meat, sugar-sweetened beverage, and time between baseline and follow up visit; furthermore, they were mutually adjusted by other color groups.

Table S5 Associations between intake of fruit and vegetable color groups at baseline and cardiometabolic risk biomarkers after ~6 years with further adjustments in HCHS/SOL, 2008-2011 to 2014-2017 (n=9 206)^a^

| % changes | | All colors^c^ | Red/Purple^d^ | White^d^ |
| --- | --- | --- | --- | --- |
|  | Original analysis | | | |
| BMI^b^, kg/m^2^ | | -0.24±0.11* |  |  |
| Post OGTT^b^, mg/dL | |  |  | -0.81±0.40* |
| Insulin^b^, pmol/L | | -0.69±0.32* |  |  |
| HDL-C^b^, mg/dL | |  | 1.59±0.80* |  |
| TG^b^, mg/dL | |  |  | -0.67±0.32* |
|  | Further adjusted by fiber | | | |
| BMI^b^, kg/m^2^ | | -0.21±0.12 |  |  |
| Post OGTT^b^, mg/dL | |  |  | -0.74±0.43 |
| Insulin^b^, pmol/L | | -0.59±0.36 |  |  |
| HDL-C^b^, mg/dL | |  | 1.64±0.81* |  |
| TG^b^, mg/dL | |  |  | -1.34±0.73 |
|  | Further adjusted by folate | | | |
| BMI^b^, kg/m^2^ | | -0.26±0.11* |  |  |
| Post OGTT^b^, mg/dL | |  |  | -0.83±0.40* |
| Insulin^b^, pmol/L | | -0.62±0.32 |  |  |
| HDL-C^b^, mg/dL | |  | 1.59±0.81* |  |
| TG^b^, mg/dL | |  |  | -1.42±0.70* |
|  | Further adjusted by vitamin A | | | |
| BMI^b^, kg/m^2^ | | -0.27±0.11* |  |  |
| Post OGTT^b^, mg/dL | |  |  | -0.84±0.40* |
| Insulin^b^, pmol/L | | -0.67±0.32* |  |  |
| HDL-C^b^, mg/dL | |  | 1.59±0.80* |  |
| TG^b^, mg/dL | |  |  | -1.47±0.70* |
|  | Further adjusted by vitamin C | | | |
| BMI^b^, kg/m^2^ | | -0.27±0.13* |  |  |
| Post OGTT^b^, mg/dL | |  |  | -0.86±0.42* |
| Insulin^b^, pmol/L | | -0.68±0.39 |  |  |
| HDL-C^b^, mg/dL | |  | 1.70±0.81* |  |
| TG^b^, mg/dL | |  |  | -1.56±0.71* |
|  | Further adjusted by vitamin E | | | |
| BMI^b^, kg/m^2^ | | -0.26±0.12* |  |  |
| Post OGTT^b^, mg/dL | |  |  | -0.81±0.41* |
| Insulin^b^, pmol/L | | -0.66±0.33* |  |  |
| HDL-C^b^, mg/dL | |  | 1.56±0.80 |  |
| TG^b^, mg/dL | |  |  | -1.43±0.70* |
|  | Further adjusted by vitamin B6 | | | |
| BMI^b^, kg/m^2^ | | -0.32±0.11** |  |  |
| Post OGTT^b^, mg/dL | |  |  | -0.85±0.41* |
| Insulin^b^, pmol/L | | -0.64±0.31* |  |  |
| HDL-C^b^, mg/dL | |  | 1.58±0.80* |  |
| TG^b^, mg/dL | |  |  | -1.39±0.71* |
|  | Further adjusted by magnesium | | | |
| BMI^b^, kg/m^2^ | | -0.24±0.12* |  |  |
| Post OGTT^b^, mg/dL | |  |  | -0.74±0.41 |
| Insulin^b^, pmol/L | | -0.40±0.32 |  |  |
| HDL-C^b^, mg/dL | |  | 1.54±0.80 |  |
| TG^b^, mg/dL | |  |  | -1.30±0.70 |
|  | Further adjusted by potassium | | | |
| BMI^b^, kg/m^2^ | | -0.24±0.14 |  |  |
| Post OGTT^b^, mg/dL | |  |  | -0.74±0.42 |
| Insulin^b^, pmol/L | | -0.38±0.38 |  |  |
| HDL-C^b^, mg/dL | |  | 1.62±0.81* |  |
| TG^b^, mg/dL | |  |  | -0.99±0.78 |

HCHS/SOL, Hispanic Community Health Study/Study of Latinos; BMI, body mass index; OGTT, oral glucose tolerance test; HDL-C, high-density lipoprotein; TG, triglycerides.

^a^Values are percentage changes in cardiometabolic risk biomarkers associated with one-serving increase in FV color groups.

^b^Cardiometabolic risk biomarkers were log-transformed due to skewed distributions and then back transformed to the estimates of coefficients on the original scale

^c^Models were adjusted for baseline data of age, sex, Hispanic/Latino heritage, field center, income, education level, whether U.S. born, years living in the U.S., medication use for hypertension and blood lipids, physical activity, sedentary behavior, smoking, alcohol use level, total energy intake, polyunsaturated fatty acids, *trans* fatty acids, whole grains, red and processed meat, sugar-sweetened beverage, time between baseline and follow up visit, plus BMI for insulin.

^d^Models were adjusted for baseline data of age, sex, Hispanic/Latino heritage, field center, income, education level, whether U.S. born, years living in the U.S., medication use for hypertension and blood lipids, physical activity, sedentary behavior, smoking, alcohol use level, total energy intake, polyunsaturated fatty acids, *trans* fatty acids, whole grains, red and processed meat, sugar-sweetened beverage, time between baseline and follow up visit, and BMI (except for BMI). Furthermore, they were mutually adjusted by other color groups.

*p-value<0.05, **p-value<0.01
